# Head injuries in professional football (soccer): Results of video analysis verified by an accident insurance registry

**DOI:** 10.1371/journal.pone.0255695

**Published:** 2021-08-11

**Authors:** Volker Krutsch, Werner Krutsch, Jonas Härtl, Hendrik Bloch, Volker Alt, Christian Klein, Claus Reinsberger, Robin Seiffert, Lorenz Huber, Johannes Weber

**Affiliations:** 1 Department of Otorhinolaryngology, Paracelsus Medical University Nuremberg, Nuremberg, Germany; 2 Department of Trauma Surgery, University Medical Centre Regensburg, Regensburg, Germany; 3 SportDocsFranken, Nuremberg, Germany; 4 Department of Sports Injury Prevention, VBG, Germany, Statutory Accident Insurance for the Administrative Sector, Hamburg, Germany; 5 Department of Sports Medicine, University of Paderborn, Paderborn, Germany; Paracelsus Medizinische Privatuniversitat - Nurnberg, GERMANY

## Abstract

**Background:**

Video analysis is one of the most commonly applied methods for analysing football injuries.

**Purpose:**

The objective of this study was to assess the accuracy of video analysis for recording head injuries in professional football from official matches in the four highest men’s professional football leagues in Germany.

**Methods:**

In this cohort study, head injuries detected by means of video analysis of all official matches over one season (2017–18) were compared to head injuries registered with the German statutory accident insurance.

**Results:**

Our video analysis yielded 359 head injuries of 287 players. The comparison of head injuries found in our video analysis to those registered with the accident insurance only yielded a match in 23.1% (n = 83), which presents a rather low verification rate. The verification rates varied between the leagues (7.0–30.8%). All injuries documented in the accident insurance registry were found in the video analysis (100%). The types of head injury most often verified by the accident insurance registry (n = 83) were contusion (43.4%), bone fractures (19.3%) and skin lacerations (18.1%). Only 66 of the 359 head injuries (18.4%) resulted in absence from at least one training session and involved a mean time loss of 18.5 days (1–87 days).

**Conclusion:**

The mismatch between the number of head injuries found in the video analysis and head injuries registered with the accident insurance is an important methodological issue in scientific research. The low verification rate seems to be due to the unclear correlation between injury severity and clinical consequences of head injuries detected by means of video analysis and the failure of football clubs to register minor head injuries with the accident insurance.

## Introduction

Football represents one of the most popular sports worldwide and has a considerable sports-specific injury risk [1, 2]. Epidemiological injury analysis in football is generally well represented in the scientific literature [2–4]. Potential injury risks and the incidence of injuries to the lower extremities are well documented [4–6]. In football, head injuries occur less frequently than injuries to the lower extremities [7]. However, the potential long-term consequences of head injuries such as neuro-psychological changes or neurodegenerative pathologies may be even more serious than injuries to the lower extremities [8–12]. Reports on the neurological and neuropsychological impact of head injuries in football are rare, and the influence of such injuries on practical routine is unclear [9]. Therefore, epidemiological head injury data are needed, particularly for professional football leagues, in which the injury burden of 2.25 to 2.46 per 1000 match hours is higher than that at lower football levels [13, 14].

In several previous studies, the registration of head injuries based on video analysis was an appropriate tool for characterising the consequences of head injuries and the need of medical treatment of football players [13, 15, 16]. Already in their video analysis of head injuries in 2018, Beaudouin et al reported typical risk factors and used public media registration of head injuries as a data base [13]. The currently used standardised video analyses [13] seem to be an appropriate method for explaining the circumstances and mechanisms of head injuries in general; yet, football injuries are still neither verified nor validated. Further information, such as the exact injury diagnosis, injury severity, absence from football or further consequences, has to be obtained from other sources. The current discussion in football about head injuries with possible neurological long-term consequences such as dementia shows the urgent need of further investigations using clear methodological structures for analysing the short-term and long-term impact of head injuries [17]. To understand the real frequency of head injuries in the different professional leagues, this study compared the results of video analyses of football matches with head injuries registered with the German statutory accident insurance ‘Verwaltungsberufsgenossenschaft’ (VBG). The purpose of this study was to investigate whether the real incidence of head injuries can be detected by means of video analysis and whether the different football leagues differ in the frequency of head injuries. A secondary purpose of this study was to obtain a more accurate number of head injuries by video analysis.

## Materials and methods

This prospective cohort study analysed head injuries by means of video material only of official matches of the four highest men’s professional football leagues in Germany (1^st^ and 2^nd^ Bundesliga, 3^rd^ and 4^th^ league). The first part of this study consisted of a standardised video analysis using publicly available video material over one season (2017–18). For the 1^st^ and 2^nd^ Bundesliga, we used video material of the national pay television channel ‘Sky’ and for the 3^rd^ and 4^th^ league an online portal account ‘die-ligen.de’, both provided by the national football association. Over one season, we documented each head injury event in every official match for which video material was available in full length. For head injuries, we used the model of critical incidents (CI) according to the criteria first published by Anderson et al (2004) and further developed by Bjoernboe et al (2014) [15, 18]. A CI was recorded if a player had sustained a hit to the head and the referee had interrupted the match, if one of the players involved in the tackling had been laying on the ground in pain for more than 15 seconds or had to be carried off the field. For correct analysis, each head injury was viewed several times at different slow-motion speeds and freeze-frames in a standardised manner. Unclear situations were immediately discussed by the study team consisting of at least two different reviewers. The reviewer team consisted of at least one medical doctor and one medical student. Both reviewers were trained in standard video analysis in football and also experienced in detecting and analysing football injuries. The rating of an incident as a head injury was followed by documenting the player’s name (number) and anthropometric data via standardised media registration (Kicker sports magazine) [6]. All recorded head injuries were entered into a secure online-based data bank termed REDCap as electronic case report forms (eCRF).

The second part of the study consisted of the verification process of each video-based head injury using the injury registration provided by the statutory accident insurance for professional athletes in Germany (VBG). This insurance covers the costs of traumatic injuries sustained by salaried athletes in training sessions and matches in German football. Head injuries are recognised as typical contact injuries and should therefore be included in the registry of the accident insurance [13, 19]. Because playing football constitutes the occupational activity of professional football players, head injuries in matches and training sessions represent occupational accidents. Team physicians of professional football clubs have to document and report each head injury to the accident insurance if a player shows any pathological symptoms during the first or any further examinations, independent of the severity or the diagnosis of the head injury. After the diagnosis of a head injury by a physician, the accident insurance must also be notified about the date of the player’s return-to-play.

The insurance company received our list of head injuries documented by means of video analysis over a complete season. The insurance company confirmed or negated the presence of a documented head injury based on our list of head injuries for each football league. To observe the ethical data regulations and protect the personal data of the players, the data on our list of 287 players were then completely anonymised by the accident insurance company. Additionally, the insurance company changed the order of players before the anonymised list was returned to our study office. This way, only the respective football level but no individual player could be retrospectively identified. The insurance company not only verified each registered head injury but also added important other information such as the correct diagnosis, the date of return-to-play, the recurrent injury rate and other relevant data. Injury collection and data sample were assessed according to the consensus statement by Fuller et al (2006) [20]. Further injuries in the period after the head injury were defined as recurrent injuries, based on the previous publication by our study group [2]. These unique study methods had been approved by the Ethics Committee of the University Regensburg (Nr. 15-101-0134) prior to the study kick-off and also met the internal regulations of the insurance company VBG.

## Statistical analysis

The frequency of head injuries was calculated with the number of Critical Incidents on the head and is shown as incidence for 1000 h match exposure. Descriptive data such as injury characteristics are presented as absolute numbers, percentages in parentheses and mean where appropriate. Data of the verification process are presented as percentages and absolute numbers. All analyses were done with Microsoft Excel®.

## Results

Overall, 359 head injuries in 287 football players of the 1^st^ and 2^nd^ Bundesliga and the 3^rd^ and 4^th^ league were documented by means of video analysis. 28 players (9.8%) sustained 2 head injuries and 13 players more than 2 head injuries (4.5%). The number of head injuries was higher in the 1^st^ and 2^nd^ Bundesliga than in the 3^rd^ and 4^th^ league. Defenders followed by midfielders sustained the most head injuries (Table 1).

**Table 1:** Anthropometric data of football players with a head injury determined by video analysis.

	1^st^ Bundesliga	2^nd^ Bundesliga	3^rd^ league	4^th^ league	Total
**Number of head injuries: n**	120	159	43	37	359
**Number of injured players: n**	88	122	42	35	287
**Mean age in years (range)**	26.6 (17–35)	27.2 (20–36)	28.3 (21–35)	25.6 (21–33)	27.0 (17–36)
**Mean height in cm (range)**	185.2 (168–199)	184.6 (171–196)	184.5 (171–196)	182.6 (170–193)	184.2 (168–199)
**Mean weight in kg (range)**	78.7 (64–97)	79.6 (61–93)	78.2 (65–87)	75.7 (60–83)	78.1 (61–97)
**Position on field: n (%)**					
**Goalkeeper**	2 (0.5%)	7 (1.9%)	1 (0.3%)	1 (0.3%)	11 (3.1%)
**Defender**	54 (15.0%)	71 (19.8%)	6 (1.7%)	11 (3.1%)	142 (39.6%)
**Midfielder**	46 (12.8%)	51 (14.2%)	12 (3.3%)	15 (4.2%)	124 (34.5%)
**Striker**	18 (5.0%)	30 (8.4%)	24 (6.7%)	10 (2.8%)	82 (22.8%)
**Football match exposure in h**	39.2	34.6	42.2	30.6	36.7

The rates of head injuries found in the video analysis and verified by the number of injuries registered with the accident insurance varied between the leagues. Mean verification rate of head injuries in the four professional football leagues was 23.1%. The highest verification rate was found for the 1^st^ Bundesliga (30.8%) followed by the 2^nd^ Bundesliga (25.2%) (Table 2). The seasonal distribution of head injuries and verified head injuries showed consistent distribution over the season.

**Table 2:** Head injuries determined by video analysis and confirmed by the accident insurance registry.

	1^st^ Bundesliga	2^nd^ Bundesliga	3^rd^ league	4^th^ league	Total
**Head injuries according to video analysis: n (%)**	120 (33.4%)	159 (44.3%)	43 (12.0%)	37 (10.3%)	359 (100%)
**Head injuries according to accident insurance registry: n (%)**	37 (44.6%)	40 (48.2%)	3 (3.6%)	3 (3.6%)	83 (100%)
**Percentage of head injuries found in video analysis and registered with the accident insurance**	30.8%	25.2%	7.0%	8.1%	23.1%

The 83 diagnosed head injuries verified by the insurance company showed contusion (43.4%), bone fractures (19.3%) and skin lacerations (18.1%) as the most common types of head injuries in professional football. The rate of concussion was 8.4% (Table 3).

**Table 3:** Diagnoses of head injuries registered with the accident insurance.

**Head injury diagnosis**	**n = 83 (%)**
**Contusion**	36 (43.4%)
**Bone fractures in total**	16 (19.3%)
** Nasal bone fracture**	7 (8.4%)
** Nasal septum fracture**	2 (2.4%)
** Orbital wall fracture**	2 (2.4%)
** Zygomatic bone fracture**	1 (1.2%)
**Skin laceration**	15 (18.1%)
**Concussion**	7 (8.4%)
**Haematoma**	3 (3.6%)
**Cervical spine (combined)**	1 (1.2%)
**Others n (%)**	5 (6.0%)

66 head injuries (18.4%) diagnosed by means of video analysis resulted in absence from sports of at least one training session (Table 4). Of these 66 head injuries, 45 (68.2%) lead to absence from one match, 6 head injuries (9.1%) to absence from two matches and 7 head injuries (10.6%) to absence from three or more matches. 8 head injuries (12.1%) lead to absence from training sessions only but not from any matches. Mean time for return-to-play after these head injuries was 18.5 days (1–87 days).

**Table 4:** Medical consequences after head injuries registered with the accident insurance.

	**Total (%)**
**Head injuries involving absence from sports: n (%)**	66/359 (18.4%)
**Recurrent injury**	374 (100%)
** head**	27 (7.2%)
** cervical spine**	6 (1.6%)
** upper extremity**	31 (8.3%)
** spine**	12 (3.2%)
** core**	4 (1.1%)
** hip**	16 (4.3%)
** thigh**	81 (21.6%)
** knee**	47 (12.6%)
** lower leg**	36 (9.6%)
** ankle joint**	62 (16.6%)
** foot**	52 (13.9%)
**Players with recurrent injuries n (%)**	**180/287 (62.7%)**
**Head injury sustained in the previous season n (%)**	14 (16.9%)

After a head injury, 180 players (62.7%) had sustained recurrent injuries (n = 374) to different body sites in the further course of the season. The rate of recurrent injury was highest for players in the 1^st^ Bundesliga with 148 injuries. The body site most affected by recurrent injury after a head injury was the thigh muscles (21.6%) followed by the ankle joints (16.6%). 7.2% of the players had sustained recurrent injury to the head; players of the 4^th^ league had the highest rate of recurrent injury to the head with 10.0% compared to the rate of other leagues of 5.2–9.5% (Table 4).

## Discussion

All head injuries verified by means of accident insurance registry were detected in the video analysis, which shows the effectiveness of video analysis in detecting head injuries. Almost 80% of head impacts detected by video analysis had not been documented in the insurance registry, which represents a large proportion of head injuries with unclear clinical impact or consequences. Potential sub-concussive head impacts may remain undetected by physicians and hence not be registered with the accident insurance company [19]. The results of this study and previous studies show that video analysis of sports injuries in professional football is still an established tool for analysing football injuries including head injuries in sports-statistical research [15, 21–23]. Previous video analyses seemed to be valuable. In addition to the questionnaire-based analysis used in recent epidemiological investigations, video analysis represents a further option to record sports injuries in football [2, 5, 19]. Video analysis has been validated and standardised for injury analysis in previous studies [15], especially in professional football, in which multiple camera views are available for each match situation. Compared to questionnaire-based analysis, video analysis additionally provides essential information about injury mechanisms on the field [13, 18]. This study assesses for the first time the currently used principles of video analysis in professional sports and shows the characteristics and potential limits of each method of injury registration [22].

Video analysis represents a further type of injury assessment in addition to the injury registry of the accident insurance, which is also well described in the literature. Previous scientific publications used accident insurance data to provide detailed knowledge about injuries in amateur and professional sports [24, 25]. The team physician report is defined as gold standard in current research [20]. With the beneficial effect of clinical injury data including symptoms, diagnostic imaging and follow-up, the accident insurance registry also provides valid information about head injuries and their outcome. The number of head injuries in professional football reported in the literature is well known, and other studies in clinical settings have shown a relevant rate of overlooked severe injuries such as concussion [14, 19]. Since only less than 30% of head injuries detected by means of video analysis had been registered with the insurance company, the discrepancy in the numbers of head injuries between the two methods is alarmingly high. Reason for this mismatch seems to be the lower severity of minor head impacts or even the underreporting of the symptoms and clinical signs of players by the medical staff [7, 26, 27]. Therefore, the accident insurance registry represents a valuable data source for recording head injuries of any given sports population. Future epidemiological surveys on injury frequency, risk, mechanisms and severity should combine both physician reports (directly or by the accident insurance) and video documentation.

In this study, 83 verified head injuries were severe injuries such as fractures or concussion, which represents a high percentage of severe injury types in this sample. It is a well-known fact that over 50% of head injuries in sports populations are overlooked and often result in neurological symptoms, and this percentage gives an impression of the real frequency of severe head injuries in daily sports [19]. The severity of potentially overlooked injuries on the head is unclear, both as a direct consequence of the injury and for consecutive recurrent injuries to other body sites. Our investigation emphasised the fact that 62.7% of players had sustained recurrent injuries after an index head injury. This alarmingly high rate of recurrent injury after head trauma leads to the assumption that head injuries may compromise the neuromotorical adaptation of players and therefore potentially facilitate consecutive injuries to other body sites [28]. The low number of 66 head injuries registered with the accident insurance in contrast to the 359 head injuries detected by means of video analysis supports the assumption that head injuries may be underreported to the accident insurance because of the potential trivialization of head injuries by medical staff. The epidemiological distribution of head injuries in this study showed a higher risk of head injuries of defenders and a consistent distribution of the frequency of head injuries over the season. Thus, seasonal attention is not relevant for preventing such injuries but educating players, especially defenders, on how to react in potential head injury situations [29]. Side-line testing by means of short questionnaires or standardised test protocols for the early detection of concussion or mild traumatic brain injuries may improve awareness of the medical staff to avoid trivialization of head injuries in football [30].

This study is the first assessment of head injuries in video analysis that is verified by accident insurance data, but the study is not without limitations. First, head injuries were only analysed over one season. The second limitation is the ethical guideline of this study and the accident insurance guidelines, which only allowed the inclusion of anonymous data. Therefore, it was not possible to carry out further sub-investigations after the blinded verification of the video analysis data by means of the accident insurance data. A further weakness of injury registration with the accident insurance already reported by Klein et al in 2019 is the lack of information on overuse and non-traumatic injuries [24]. Scientific evidence consists of physician reports and video analysis [1, 15]. Because physicians working with athletes should read scientific injury statistics critically, this study shows that they need to know that differences in epidemiological injury analyses depend on methodological considerations. Additionally, comparing video analysis between different professional football leagues is not without bias because of the higher quality and quantity of video footages in the two highest leagues than in the lower leagues. Future injury investigations should focus on head injuries, especially in the case of sub-concussive injuries [19, 31]. For the practical routine in professional football, use of video footage could become common practise to provide further information on injuries. A detailed video review by the team physician, tunnel doctor or a video referee during the match seems to be an appropriate method for detecting head injuries. If the detection of head injuries can be improved by video analysis, this method may also become more accepted by coaches and managers. In professional football, training sessions are also analysed by means of video material; however, such analyses are less frequently used for medical investigations than for athletic or tactical improvement. Changing this aspect may also further optimize the prevention of head injuries.

## Conclusion

The mismatch in the frequency of head injuries between video analysis and insurance registry shows the weakness of current head injury registration and the possible underreporting by medical staff. The inability of video analysis to indicate the severity and clinical consequences of a head injury and the failure of clubs to register minor head injuries with the accident insurance seem to be probable factors for the discrepancy between the two assessment methods. Especially the rate of head injuries underreported to the accident insurance registry is remarkable. Further scientific investigations should focus on this topic to strengthen awareness when evaluating head injuries. Because head injuries are caused by typical contact mechanisms, the accident insurance registry seems to be an appropriate tool for addressing the accurate number of head injuries. Further research is necessary to investigate valid injury analysis for head injuries, especially in the case of sub-concussive impacts.
